# Functional properties of food packaging solutions alternative to conventional multilayer systems

**DOI:** 10.1007/s13197-024-06038-5

**Published:** 2024-07-30

**Authors:** Anna Mengozzi, Daniele Carullo, Francesca Bot, Stefano Farris, Emma Chiavaro

**Affiliations:** 1 Department of Food and Drug, University of Parma, Parco Area delle Scienze 27/A, Parma, 43124 Italy; 2 DeFENS, Department of Food, Environmental and Nutritional Sciences, University of Milan, via Celoria 2, Milan, I-20133 Italy

**Keywords:** Food packaging, Biopolymers, Barrier properties, Optical properties, Modified atmosphere

## Abstract

This study aimed to investigate the most important functional properties of multilayer and alternative packaging with improved sustainability specifically used for modified atmosphere (MAP) and chilled food products. A multilayer material with a thickness reduction, mono-PET, paper/PE-EVOH-PE, and a biopolymer for trays, together with a SiO_x_-coated PET, and a cellulose/PLA-based for lids were analyzed for their optical, tensile, and gas-vapor barrier properties, which were compared to those of conventional trays/lids (PET-EVOH-PE multilayer structures). All the alternative solutions showed good UV-light screening ability, together with high transparency in the visible range, and tensile properties greater than those displayed by conventional configurations. Lid alternative materials exhibited a significantly higher performance in terms of oxygen and water vapor barrier properties as compared to that displayed by conventional counterparts. The tray alternative solutions performed better than the conventional ones against CO_2_ and O_2_ permeation, with values lower than the detection limit of the instrument (0.01 cm^3^ m^− 2^ day^− 1^ and 0.25 cm^3^ m^− 2^ day^− 1^ for O_2_ and CO_2_, respectively). This study demonstrated the high potential of alternative packaging in replacing the current materials intended for storing highly perishable foods stored under MAP and cold storage.

## Highlights

Functional properties of different packaging systems were investigated.

All tested materials exhibited good transparency and UV shielding properties.

Tensile properties of mono-material and conventional tray films were comparable.

Alternative materials showed higher barrier properties than conventional solutions.

Alternative solutions have the potential to replace conventional configurations.

## Introduction

Fostered by convenience, functionality, and excellent quality and safety, the consumption of packaged foods has increased significantly over the last decades (Kan and Miller 2022; Asgher et al. 2020). The global packaged food market accounted for $ 1.9 trillion in 2020 and is bound to reach a value of $ 3.4 trillion by 2030 (Kan and Miller 2022). Hence, packaging plays a crucial role in the food industry, providing quality preservation and safety maintenance of food products (McMillin 2017). The main goal of packaging is to protect food from deterioration due to biological (e.g., microbial spoilage, and enzymatic reactions) and physicochemical factors (e.g., gas transfer, moisture loss/uptake, and mechanical stresses), while facilitating all the logistic phases from the company to the consumer’s home (Asgher et al. 2020). However, nowadays food packaging waste represents an important issue that must be addressed to possibly tackle the environmental challenges caused by improper waste management (Kan and Miller 2022). In Europe, petroleum-based plastics are mostly used to produce food packaging in both rigid and flexible configurations (Kan and Miller 2022). Plastic polymers are usually combined to create multilayer packaging systems endowed with excellent functional features, such as mechanical strength, barrier properties, and heat sealability, but also low cost (Bauer et al. 2021). Nevertheless, heterogeneous multilayer plastics are not environmentally sustainable: their short service life and their non-renewable origin, together with the high volume of waste generated, pose serious risks to the environment (Asgher et al. 2020). In addition, the combination of materials with different chemical compositions, such as polyethylene terephthalate (PET) and polyolefins like polyethylene (PE) or polypropylene (PP), is one of the main causes complicating their mechanical recycling, together with the difficult identification, collection, and separation of the different plastic layers within current recycling plants (Kaiser et al. 2017).

Therefore, considering the increasing demand for packaged food products worldwide, there is an urgent need to switch to alternative packaging materials able to ensure food quality and safety similar to conventional multilayer packaging, but with a reduced environmental impact (Kan and Miller 2022). In this scenario, the outlined strategies of the European Commission aiming for a green transition in packaging development encompass (i) the reduction of over-packaging by decreasing the overall thickness and unnecessary packaging, (ii) the reduction of packaging complexity by using easily recyclable materials (e.g., mono-materials or recycled materials), and finally (iii) the use of bio-based and/or biodegradable/compostable materials (European and Union 2018; European Commission 2018). Hence, replacing heterogeneous multilayer plastic packaging with materials and configurations that allow for over-packaging reduction, increase the recycling rate, and reduce the upstream amount of plastics of fossil origin can represent a viable strategy to fulfill a circular economy approach in the food packaging sector (Bauer et al. 2021; Kaiser et al. 2017).

Several packaged foods rely on modified atmosphere packaging (MAP) to maintain safety and extended shelf-life (McMillin 2017). MAP techniques have been used on a wide range of fresh or chilled foods, including raw and cooked/processed meat, fish and poultry, fresh pasta, fresh and cut fruits and vegetables, as well as coffee, tea, and confectionary products (Goswami and Mangaraj 2011). Moreover, foods stored under MAP require highly efficient packaging materials in terms of barrier properties since the gas/vapor permeability of the package may alter the internal atmosphere (Langhe and Ponting 2016).

The packaging solutions employed for the storage of chilled food products under MAP involve a two-component lid/tray sealed system generally made of different plastic layers, such as PET, PE, linear low-density polyethylene (LLDPE), and polyamide (PA). The latters are often coated with barrier coatings, e.g., ethylene vinyl alcohol (EVOH), aluminum oxide (AlO_x_), and silicone oxide (SiO_x_), to maximize the barrier performance, thus limiting any gas exchange across the packaging material and preserving the modified atmosphere over storage (Galikhanov et al. 2015; McMillin 2017; Schneider et al. 2009; Korte et al. 2023). In particular, for foods sensitive to oxygen-dependent decay mechanisms (microbiological spoilage, lipid oxidation, and discoloration), MAP must provide and maintain an anoxic environment either by using passive systems (e.g., high oxygen barrier materials) or active devices (e.g., oxygen scavengers) (Langhe and Ponting 2016).

Nowadays, there are only a few alternative packaging solutions for chilled food under MAP aimed at improving sustainability at the same level of protection granted by multilayer configurations (Korte et al. 2023). One example is given by paper-based trays or pouches, intended for refrigerated sliced meat and cheese products or fresh vegetables, which can be sorted in the paper stream collection in Europe (McMillin 2017).

Based on the above considerations, in this work, a comparative performance analysis between conventional packaging materials (both tray and lid films) and different alternative solutions reliant on plastic weight reduction, use of potentially recyclable packaging such as mono materials (i.e., mono-PET and paper), and bio-based materials was executed. To this end, optical (transparency, and haze), mechanical (elastic modulus, elongation at break, and tensile strength), and barrier (carbon dioxide, oxygen, and water vapor transmission rates) properties of tested materials were assessed. The outcomes of this work will help in supporting further innovations in the development of MAP systems for chilled foods, also considering the increasing requirements for high sustainability, food safety, and quality imposed by European legislation.

## Materials and methods

### Packaging materials and thickness measurement

The different commercial configurations of both trays and lids (Table 1) were gently provided by different packaging companies based in EU and were selected for this study due to their specific application in chilled food products stored under MAP (e.g., cured ham, cheese).

**Table:**
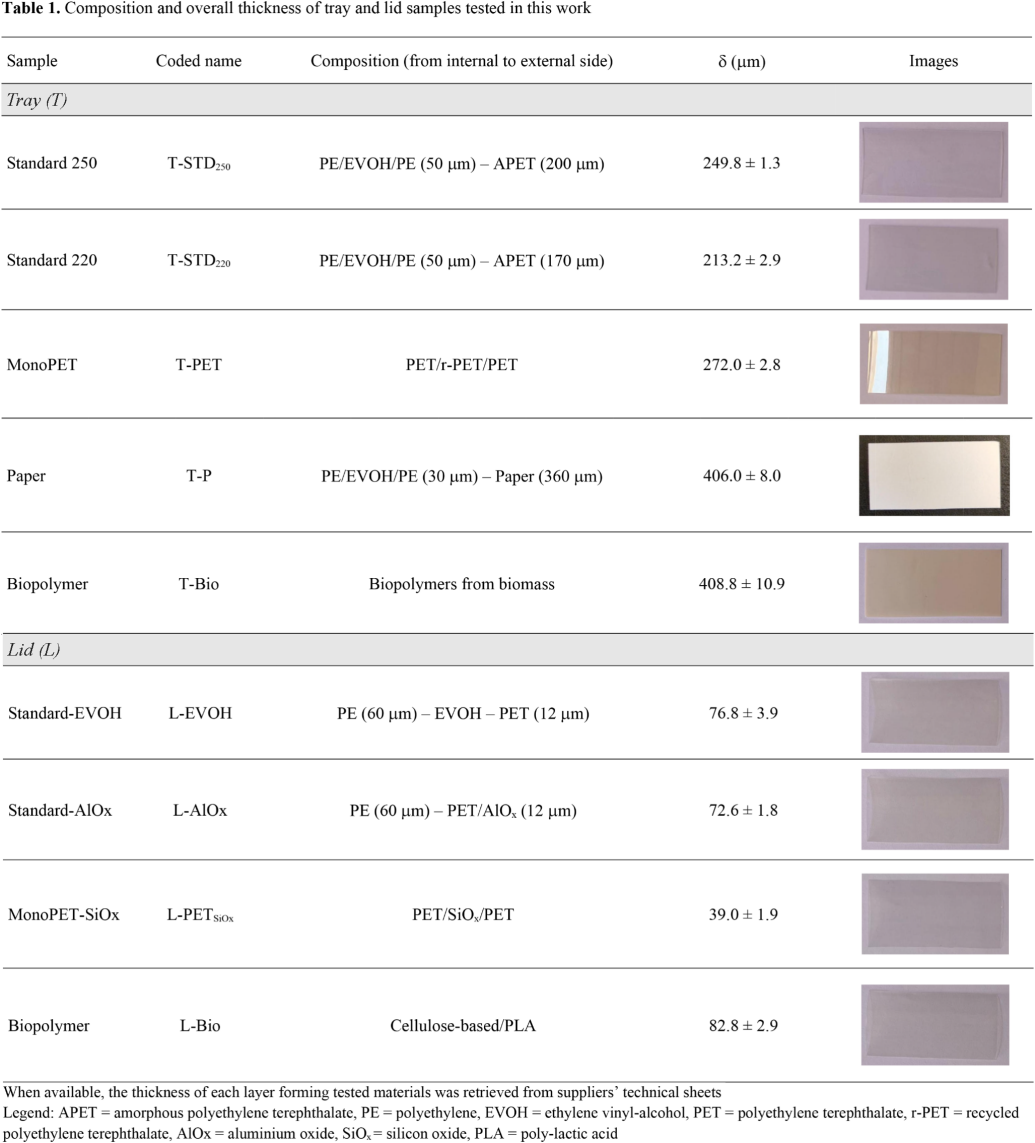

The conventional solution for the tray, coded as T-STD_250_, consisted of a coextruded amorphous polyethylene terephthalate (APET) film of 200 μm thickness with an oxygen/water vapor barrier PE/EVOH/PE structure. Four alternative configurations were also investigated, namely (i) the same coextruded material as that previously described, but with a 15% thickness reduction on the APET layer (T-STD_220_); (ii) a coextruded three-layer mono-material, coded as T-PET, based on both virgin and recycled PET (r-PET), (iii) a laminated multilayer material (T-P) made of a 360 μm thick paper sheet with a PE-EVOH-PE system, and (iv) a coextruded bio-based mono-material (T-Bio) made by a specific type of polyester obtained upon polycondensations of diacids and diol (confidential info).

In the case of the lids, coextruded standard configurations (i.e., L-EVOH and L-AlO_x_) involved a layer of either EVOH or AlO_x_ onto a 12 μm-thick PET film, with the latter being further combined with a 60 μm-thick PE film. Once again, two alternative solutions were scouted, such as a coextruded SiO_x_-treated PET (L-PET_SiOx_), and a laminated bio-polymeric material (L-Bio) coupling cellulose and polylactic acid (PLA).

The thickness (δ, in µm) of tray and lid films was measured employing a digital micrometer (Dialmatic DDI030M, Bowers Metrology, Bradford, UK) with an accuracy of 1 μm at 15 different random locations. Finally, the averaged thickness was considered for all executed measurements (Farris et al. 2009).

### Analytical determinations

#### Optical properties

Transparency (T_550_, in %) and haze (H, in %) of all the investigated packaging materials were evaluated through a high-performance UV-Vis spectrophotometer (Lambda 650, PerkinElmer, Waltham, MA, USA), capable of scanning within a broad wavelength range of 190–900 nm. Specifically, T_550_ was measured following the ASTM D1746 in terms of specular transmittance, obtained when the transmitted radiant flux includes only the light transmitted in the same direction as that of the incident flux at 550 nm. Such wavelength is usually chosen to compare the transparency of samples at conditions to which human eyes are highly sensitive (Farris et al. 2010).

On the other hand, the haze was determined according to the ASTM D1003 standard within the wavelength range of 380–780 nm, using a 150 mm integrating sphere that allowed to trap also the diffused transmitted light. Haze is defined as the scattering of light by a specimen responsible for the reduction in contrast of objects viewed through it and indicates the percentage of incident light that deviates by more than 2.5° through the specimen from the original direction of the incident light. Low haze values are associated with high clarity of the materials (Farris et al. 2009). For both transparency and haze, the final data are collected by averaging among a triplicate of analyses.

To investigate the UV-Vis transmission properties of tested samples, transmittance spectra were also captured in the wavelength region of 200–800 nm.

#### Mechanical properties

The elastic modulus (E, in MPa), elongation at break (EAB, in %), and tensile strength (TS, in MPa) of the different materials were obtained by tensile tests using a Z005 dynamometer (Zwick Roell, Ulm, Germany), coupled to the software TestXpert V10.11 for data elaboration. Following the ASTM D882 standard method, film strips of 15 cm in length and 2.5 cm in width were mounted between two clamps 10 cm apart and tested using a 5 kN load cell and a crosshead speed varying between 50 and 500 mm/min depending on the elongation of the specimens. Each average value has been calculated from at least 5 replicates.

#### Gas and water vapor barrier properties

Measurements of carbon dioxide, oxygen, and water vapor barrier properties of tested materials were executed on a 50 cm^2^ surface via a TotalPerm permeability analyzer (ExtrasolutionSrl, Capannori, Italy) equipped with an electrochemical sensor for oxygen detection and an infrared sensor for carbon dioxide and water vapor detection, respectively. The XS-Pro software (Extrasolution Srl, Capannori, Italy) was used for data acquisition and analysis.

The carbon dioxide transmission rate (CO_2_TR, in cm^3^ m^–2^ day^–1^) and oxygen transmission rate (O_2_TR, in cm^3^ m^–2^ day^–1^) were determined at 23 °C and 50% relative humidity (RH) according to the ASTM F2476 and ASTM F2622, respectively. According to the isostatic method, a constant partial pressure difference between the two semi-chambers of the permeation cell of 1 atm was kept throughout the analysis, with a nitrogen carrier flow of 10 mL min^− 1^. The water vapor transmission rate (WVTR, in g m^–2^ day^–1^) was determined using the standard method ASTM F1249, again with a nitrogen flow of 10 mL min^–1^, at 38 °C and 90% RH (tropical conditions). All the experiments were performed by placing the external side of each sample towards the upper semi-chamber, where the humid test gas (i.e., oxygen, and carbon dioxide) was flushed. Only for the cellulose/PLA sample, specimens were masked using an aluminum-tape mask at the edges to avoid lateral permeation through the fibrous network (Rovera et al. 2020). Each CO_2_TR, O_2_TR, and WVTR value is derived from at least three analyses (Carullo et al. 2023).

### Statistical analysis

The statistical significance of differences in the properties and behavior of packaging films was determined by one-way analysis of variance (ANOVA) using the SPSS 27 software (SAS, Cary, NC). When significant differences were found, Tukey’s post-hoc test was used to detect significant differences at *p* < 0.05 in case of equal variances. Dunnett’s T3 test was used when the variances were not equal.

## Results and discussion

### Optical properties of packaging materials

The optical properties of food packaging are of utmost importance as they allow one to see through the wrapping, thus showing the appearance of the food. This indicator is known to drive consumers’ purchasing choices (Farris et al. 2009). In the specific case of chilled food products, packaging materials must be highly transparent, whilst sheltering from specific light wavelengths that may trigger oxidation and discoloration phenomena (Baele et al. 2021; Domínguez et al. 2019).

**Fig. 1:**
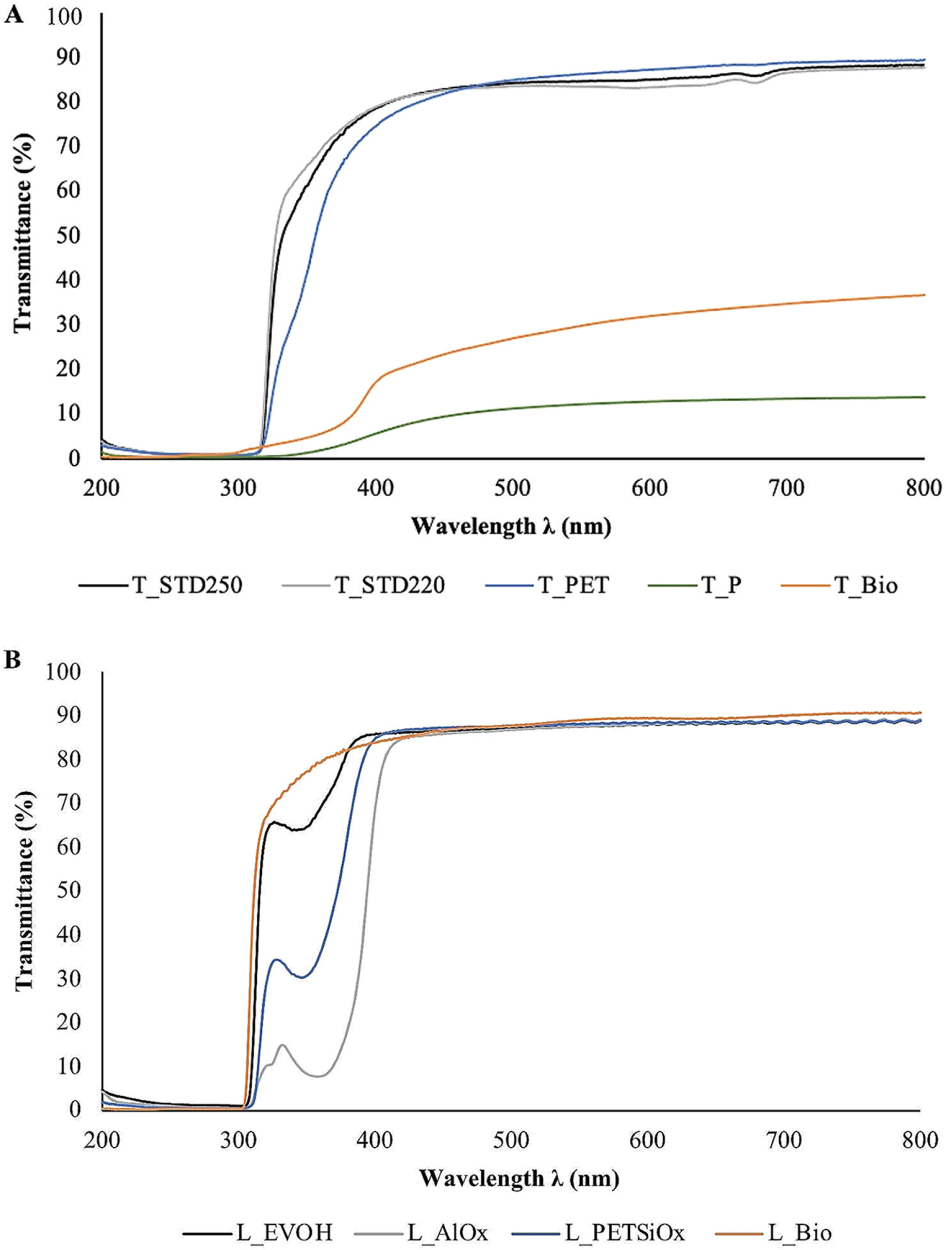
UV-Vis transmission spectra of the trays (**A**) and lids (**B**) packaging materials tested in this work

Figure 1 shows the UV-Vis transmission spectra (200–800 nm) for both tray and lid films. All the tested packaging materials were endowed with a UV-shielding behavior as they displayed a drop in light transmission below 400 nm. Similar UV-light spectra were retrieved by Jakobsen et al. (2005) who dealt with the characterization of APET-PE films destined for cured meat. In our case, a sharp decrease in the material transmittance at around 400 nm until reaching a value close to zero in the UV-C region (100–280 nm) was observed. This behavior is attributed to the aromatic ring and the carbonyl group of PET, which blocks the penetration of wavelengths below 315 nm (Curtzwiler et al. 2017). Such a trend was more pronounced for those materials containing a PET layer, including T-STD_250_, T-STD_220_, T-PET, L-EVOH, L-AlO_x_, and L-PET_SiOx_ (Fig. 1).

Concerning the transparency analysis (Table 2), T-STD_250_, T-STD_220,_ and the T-PET tray films exhibited the highest transmittance values within the visible region (84% < T_550_ < 86%), thus indicating a moderate degree of transparency. These results are mainly ascribable to the high clarity of amorphous PET, being the main component of the abovementioned plastic tray films (Nisticò 2020). In agreement with our results, Lim et al. (2021) found the transparency of trays mainly composed of PET to be approximately 86%. On the other hand, the paper and biopolymer-based tray films are characterized by very low transmittance values at 550 nm (12% and 29%, respectively), owing to the intrinsic opacity of the films. These poor optical properties are also confirmed by the high haze values (92.11% and 97.70% for T-P and T-Bio, respectively), which exceeded the 30% threshold value for light diffusion (Chatterjee et al. 2014). However, the lid films behaved decidedly better, with transparency values between 87% and 89%. This has great practical importance since the top view of a package is, in most cases, the dominant one at the retailers. Noteworthy, all the films include a barrier layer, i.e. EVOH, AlO_x_, and SiO_x_, which does not affect the transparency as already observed by Bauer et al. (2021).

**Table 2:** Values of the transparency (T_550_) and haze (H) for the packaging materials tested in this work

Material	T_550_ (%)	H (%)
*Tray*		
T-STD_250_	84.7 ± 0.1^b^	13.4 ± 0.7^b^
T-STD_220_	83.4 ± 0.3^b^	14.0 ± 0.8^b^
T-PET	86.2 ± 0.1^a^	4.7 ± 0.2^c^
T-P	12.1 ± 0.4^d^	92.1 ± 7.0^a^
T-Bio	29.8 ± 1.1^c^	97.7 ± 1.6^a^
*Lid*		
L-EVOH	87.5 ± 0.2^b^	9.9 ± 0.6^b^
L-AlOx	87.6 ± 0.1^b^	14.1 ± 1.0^a^
L-PET_SiOx_	88.0 ± 0.1^b^	9.2 ± 0.3^b^
L-Bio	88.9 ± 0.3^a^	6.6 ± 0.3^c^

^a, b, c, d^ For each parameter and packaging type (tray or lid), different letters within the same column denote significant differences (*p* < 0.05) among samples

### Tensile properties of packaging materials

Resistance to tearing, vibration, shocks, compression/crushing, and good machine handling features play a vital role in keeping package integrity throughout the food supply chain. In particular, stiffness, flexibility, and toughness are strictly sought after in semi-rigid thermoformed tray solutions for food applications (Buntinx et al. 2014).

The results of tensile tests carried out on selected packaging materials both in transverse and machine directions (TD and MD, respectively) are reported in Table 3. The plastic multilayer films exhibited on average an elastic modulus of 1900 MPa in both machine and transverse directions. Nevertheless, the T-PET film showed a significantly (*p* < 0.05) higher elastic modulus (2289 and 2250 MPa for transverse and machine direction, respectively) as compared to the other multilayer films, owing to the absence of weak PE layers and, hence, displaying good toughness. Good mechanical properties were recorded for the paper-based film due to the coupling with a PE-EVOH-PE layer (Shorey and Mekonnen 2022). However, statistical differences (*p* < 0.05) within the paper-based film were highlighted in terms of elongation at break and tensile strength between the tested directions (39.7% and 49.4 MPa in TD vs. 13.7% and 33.3 MPa in MD, respectively). As expected, the bio-based polymer tray was characterized by the greatest elastic modulus, as well as by the lowest elongation at break (Table 3), due to its high rigidity, brittleness, and reduced degree of plasticity pertaining to biobased polyesters (De Beukelaer et al. 2022). These characteristics may potentially lead to the formation of discontinuities, cracks, or even large breakages upon mechanical stresses (e.g., shocks, vibration, and compression/crushing) likely occurring during the processing and transportation phases (Pietrosanto et al. 2020).

**Table 3:** Values of elastic modulus (E), elongation at break (EAB), and tensile strength (TS) in both transverse (TD) and machine direction (MD) for the packaging materials tested in this work

Material	E (MPa)	EAB (%)	TS (MPa)
*Tray – TD*			
T-STD_250_	1982 ± 58^c^	^*^5.0 ± 2.7^b^	50.2 ± 1.4^a^
T-STD_220_	1828 ± 28^b^	3.8 ± 0.2^ab^	49.6 ± 1.4^a^
T-PET	2289 ± 43^d^	15.0 ± 2.0^c^	58.3 ± 2.7^b^
T-P	1604 ± 62^a^	^*^39.7 ± 1.7^d^	^*^49.4 ± 6.9^a^
T-Bio	4544 ± 226^e^	2.5 ± 0.1^a^	45.2 ± 2.8^a^
*Tray – MD*			
T-STD_250_	1900 ± 76^A^	^*^10.5 ± 1.2^B^	51.5 ± 3.1^C^
T-STD_220_	1851 ± 18^A^	3.8 ± 0.2^A^	49.6 ± 1.4^C^
T-PET	2250 ± 26^B^	16.0 ± 3.9^B^	59.9 ± 1.8^D^
T-P	1745 ± 105^A^	^*^13.7 ± 3.3^B^	^*^33.3 ± 1.0^A^
T-Bio	4082 ± 130^C^	2.2 ± 0.2^A^	40.9 ± 1.3^B^
*Lid – TD*			
L-EVOH	1343 ± 152^a^	39.9 ± 7.0^bc^	37.8 ± 2.0^a^
L-AlO_x_	1166 ± 39^a^	45.4 ± 4.1^c^	39.2 ± 1.7^a^
L-PET_SiOx_	4575 ± 488^c^	29.5 ± 7.6^a^	133.8 ± 17.9^c^
L-Bio	2817 ± 347^b^	^*^33.6 ± 5.6^ab^	63.8 ± 2.3^b^
*Lid – MD*			
L-EVOH	1356 ± 43^A^	47.5 ± 7.2^BC^	37.8 ± 1.1^A^
L-AlO_x_	1090 ± 62^A^	53.2 ± 9.1^C^	39.4 ± 2.0^A^
L-PET_SiOx_	4698 ± 144^C^	40.0 ± 7.1^B^	135.3 ± 8.8^C^
L-Bio	2998 ± 337^B^	^*^7.2 ± 3.9^A^	65.5 ± 2.6^B^

^a, b, c, d^ For each parameter and packaging type (tray or lid), different lowercase and uppercase letters within the same column denote significant differences (*p* < 0.05) among samples when analyzed in TD and MD, respectively. When reported, the symbol * denotes a significant difference (*p* < 0.05) between TD and MD within a same material

As far as the lid films are concerned, L-EVOH and L-AlO_x_ multilayer systems showed comparable (*p* > 0.05) mechanical properties, with a percentage of elongation at break ranging between 39% and 47%. Interestingly, similar values of E, EAB, and TS were disclosed by Carullo et al. (2023) when characterizing three multi-layer systems currently commercialized for food packaging purposes. This pinpoints that L-EVOH and L-AlO_x_ structures have “acceptable” mechanical properties, that is, they are suitable to undergo industrial applications. The L-PET_SiOx_ film showed the highest value of E, together with extensibility ranging between 29% and 39% in TD and MD, respectively. The high strength of the above sample is surely imparted by the rigidity of the silicon oxide layer (E_metal_ ≈ 80 GPa) that, in turn, curbs flexibility (Howells et al. 2008; Galotto et al. 2008). Such toughness can be also observed in the high tensile strength values (133.8 MPa and 135.3 MPa in TD and MD, respectively) in conventional lids (*p* < 0.05). Alike tray films, the biopolymer lid had a higher E and significantly lower elongation at break in MD (7%) as compared to the conventional materials (*p* < 0.05), likely due to the PLA layer which provides brittleness and rigidity to the material (Pietrosanto et al. 2020).

### Barrier properties of packaging materials

The barrier properties of packaging materials to gases and moisture are known to affect the quality of food items throughout storage (Bauer et al. 2021). Most of the products with MAP require an atmosphere devoid of oxygen to prevent lipid oxidation, color/flavor instability, and microbial spoilage (Langhe and Ponting 2016). Therefore, the associated packaging material must display adequate barrier properties that reduce/minimize gas exchange. Table 4 shows the CO_2_TR, O_2_TR, and WVTR of the investigated samples. Regarding the CO_2_ barrier properties, T-Bio showed the lowest value as compared to the standard multilayer film, which suggests the presence of a barrier layer within the biopolymer tray film structure. Significantly (*p* < 0.05) different O_2_TR values were instead detected when dealing with the two multilayer conventional plastic films. The excellent oxygen barrier properties (< 1 cm^3^ m^− 2^ day^− 1^) belonging to these samples are imparted by the EVOH layer sandwiched between the two PE layers, being the most commercially employed material when high oxygen sheltering effects are required (Bauer et al. 2021; Farris et al. 2009). This agrees with the performances already reported in the literature for multilayer plastic films mainly made of APET and PE (Buntinx et al. 2014). Finally, it is interesting to note that T-STD_220_, T-PET, and T-Bio exhibited similar WVTR values (*p* > 0.05). However, the paper tray film (T-P) showed a significantly (*p* < 0.05) higher value (25.80 g m^− 2^ day^− 1^) in comparison to all the other materials. This can be attributed to the porosity of the paper-based layer which favors the diffusion of vapor, even though the presence of hydrophobic polyolefins (e.g., double PE layer) already guarantees good moisture barrier properties (Carullo et al. 2023).

**Table 4:** Values of carbon dioxide transmission rate (CO_2_TR), oxygen transmission rate (O_2_TR), and water vapor transmission rate (WVTR) for the packaging materials tested in this work

Material	CO_2_TR (cm^3^ m^− 2^ day^− 1^)	O_2_TR (cm^3^ m^− 2^ day^− 1^)	WVTR (g m^− 2^ day^− 1^)
*Tray*			
T-STD_250_	1.53 ± 0.17^b^	^*^0.33 ± 0.06	< LDL
T-STD_220_	4.67 ± 0.52^c^	^*^0.78 ± 0.09	3.97 ± 0.33^a^
T-PET	< LDL	< LDL	3.96 ± 0.27^a^
T-P	< LDL	< LDL	25.80 ± 1.92^b^
T-Bio	0.53 ± 0.10^a^	< LDL	2.85 ± 0.27^a^
*Lid*			
L-EVOH	4.32 ± 0.32^a^	0.11 ± 0.02^a^	4.97 ± 0.58^b^
L-AlO_x_	18.19 ± 1.17^b^	3.18 ± 0.36^b^	1.51 ± 0.20^a^
L-PET_SiOx_	3.20 ± 0.38^a^	0.49 ± 0.07^a^	0.86 ± 0.11^a^
L-Bio	19.74 ± 1.42^b^	< LDL	2.18 ± 0.21^a^

^a, b, c, d^ For each parameter and packaging type (tray or lid), different letters within the same column denote significant differences (*p* < 0.05) among samples. When reported, the symbol * denotes a significant difference (*p* < 0.05) with a Student’s t-test when comparing only two samples

Legend: LDL = lower detection limit (0.25 cm^3^ m^− 2^ day^− 1^ for CO_2_TR, 0.01 cm^3^ m^− 2^ day^− 1^ for O_2_TR, and 0.0022 g m^− 2^ day^− 1^ for WVTR).

L-EVOH and L-PET_SiOx_ showed comparable CO_2_TR and O_2_TR values (*p* > 0.05), thus clearly indicating that both barrier layers (i.e., EVOH and SiO_x_) impaired the transport of carbon dioxide and oxygen across the lidding films (Korte et al. 2023). Together with EVOH, silicon oxide (SiO_x_) coatings can successfully boost the gas barrier properties of bare plastic packaging, owing to a 100-fold reduction in the permeation of gases through polymer film (Howells et al. 2008). L-AlO_x_ and L-Bio lid films have similar CO_2_TR values, despite being both significantly higher (*p* < 0.05) as compared to L-EVOH and L-PET_SiOx_. L-AlO_x_ showed a good performance in terms of oxygen and moisture barrier properties, confirming the barrier capacity of the AlO_x_ layer (Galikhanov et al. 2015; Butler and Morris 2016). The comparison among samples concerning the WVTR revealed that all lid materials performed well with very similar values, except for the L-EVOH film lid, which was characterized by a significantly (*p* < 0.05) higher value (4.97 g m^− 2^ day^− 1^). Overall, the best performance in terms of gas/water vapor transmission rates was shown by the L-PET_SiOx_ sample, which had the lowest CO_2_TR, O_2_TR, and WVTR (Table 4).

## Conclusions

This study highlighted the great potential of alternative packaging solutions to replace conventional multilayer configurations for MAP chilled food products as far as their functional properties are concerned, thus aligning with the rising demand for greater sustainability and encompassing factors like reduced weight, enhanced recyclability, and utilization of bio-based materials.

All the alternative packaging configurations tested in this work showed good transparency and UV-shielding behavior, as well as comparable or superior mechanical/gas-vapor barrier properties to those of conventional solutions. Future studies will focus on the effectiveness of the tested alternative packaging systems to guarantee the required shelf-life of MAP chilled food products as compared to conventional solutions and will provide a quantitative assessment of their environmental sustainability through a life cycle assessment (LCA) analysis.

## Data Availability

The data that support the findings of this study are available from the corresponding author upon reasonable request.
